# Applicability of a previously validated readmission predictive index in medical patients in Singapore: a retrospective study

**DOI:** 10.1186/1472-6963-13-366

**Published:** 2013-09-29

**Authors:** Shu Yun Tan, Lian Leng Low, Yong Yang, Kheng Hock Lee

**Affiliations:** 1 Department of Family Medicine and Continuing Care, Singapore General Hospital, Bowyer Block A, Level 2, Outram Road, 169608 Singapore, Singapore; 2 Department of Epidemiology, Medical Board, Singapore General Hospital, Singapore, Singapore

**Keywords:** Unplanned readmission, LACE, Emergency department, Singapore

## Abstract

**Background:**

Hospital readmissions are serious and costly events, and readmission rates are considered to be an indicator of quality in health care management. Several models to identify patients at risk of unplanned readmissions have been developed in Western countries, but little is known about their performance in other countries. This paper reports the possible utility of one such model developed in Canada, the LACE index, in patients in a tertiary hospital in Singapore.

**Methods:**

We used administrative data from Singapore General Hospital for patients admitted between 1st January 2006 and 31st December 2010. Data such as demographic and clinical data including disease codes were extracted. The patient cohort was divided into two groups with a LACE index of 10 as the cutoff. Multivariate logistic regression analysis models were used to compare the outcomes between the two groups of patients with adjustment for age, sex, ethnicity, year of discharge, intensive care unit admission, and admission ward class.

**Results:**

Overall, 127 550 patients were eligible for analysis. Patients with a LACE index ≥ 10 had a higher risk of 30-day unplanned readmission after index discharge (odds ratio [OR]: 4.37; 95% confidence interval [CI]: 4.18-4.57). After adjustment, the risk remained significant (OR: 4.88; 95% CI: CI 4.57-5.22). The C-statistic for the adjusted model was 0.70 (P < 0.001). Similar results were shown for 90-day unplanned readmission and emergency visits after the same adjustment.

**Conclusion:**

The use of the LACE index may have significant application in identifying medical patients at high risk of readmission and visits to the Emergency Department in Singapore.

## Background

Hospital readmissions are serious and costly events, which are potentially preventable [1,2]. The hospital readmission rate is widely considered an indicator of quality in health care management. In the United States, one study reported that as many as 21% of medical patients were readmitted to hospital within 30 days of discharge. The cost of such unplanned readmission was estimated at USD 17.4 billion in 2004, contributing to almost 20% of all hospitalization costs [2].

Singapore is an island nation of 5.4 million inhabitants [3]. Like many developed and developing countries, Singapore faces the challenges of a rapidly ageing population with demand for hospital beds exceeding supply [4,5]. There is a need to identify patients who have a higher use of hospital resources to improve the effectiveness of health care systems. One of the strategies to identify this group of patients is to develop a risk model to identify those at high risk of readmission. Many models have been developed to predict the risk of hospital readmissions [6-8], but most were developed in Western countries such as the United States, United Kingdom and Canada. We were interested in testing the applicability of one such model, the LACE index (See the ‘Components of LACE index’) [7], in a hospital setting in Singapore. We found that the variables used in the LACE index (**L**ength of stay, **A**cute (unplanned) readmission, **C**omorbidity burden and number of **E**mergency visits in the last 6 months) could be extracted from our existing electronic medical records. Moreover, the index has been shown to have acceptable performance (C-statistic 0.68; 95% confidence interval (CI): 0.65–0.71), indicating that it may have practical clinical utility. The LACE index ranges from 0 to 19, with a higher index indicating a greater risk for 30-day readmission or death. A further analysis found that a LACE cutoff of 10 was able to discriminate patients into low and high risk for readmission, with a two-fold higher readmission risk and mortality risk in the high-risk group [9].

Components of the LACE index

**L:** Length of hospital stay. Number of days between admission to and discharge from acute care hospital for the index hospitalization

**A:** Acuity on admission. Rating of need for care at time of index admission: unplanned (acute) or elective (non-acute)

**C:** Comorbidity. Number of co-existing medical conditions at the time of index hospitalization as measured by Charlson score with updated disease category weights

**E:** Emergency department visits. Number of emergency department visits made in the 6 months before the index hospitalization

We conducted a study to examine the ability of the LACE index to discriminate medical patients with higher risk for readmission in Singapore. Our hypothesis was that patients with LACE ≥ 10 had a higher rate of unplanned readmissions. We limited our study to patients admitted to medical wards in our hospital to allow for a more homogenous group of patients and also to allow comparison with a study carried out in Canada using the LACE index [9].

## Methods

### Setting

Our study was conducted in Singapore General Hospital (SGH), the largest tertiary care hospital in Singapore. It has 29 clinical departments, many of which are established as national referral centers. There are 1 600 beds, and care is provided for approximately 80 000 inpatients per year serving approximately one-quarter of Singapore’s population [10].

### Study population

We selected patients aged ≥ 21 years who were admitted to SGH between January 2006 and December 2010 (n = 132 861). We focused on patients admitted to the medical departments (Cardiology, Endocrinology, Dermatology, Gastroenterology, Geriatric, Hematology, Internal Medicine, Neurology, Oncology, Renal, Respiratory, and Rehabilitation Medicine). We designated the first hospital admission during the study period as the index hospitalization. Patients with subsequent in-hospital deaths (n = 1 848), patients with index hospitalization in psychiatry wards (n = 644), and patients discharged to nursing homes or other intermediate–long-term care facilities (n = 2 819) after index hospitalization were excluded from the study, because the focus of the study was on patients who were discharged to their homes. After exclusion, 127 550 patients were eligible for analysis.

### Data collection and variables

We extracted information from an administrative electronic database maintained by the Department of Information Technology, Singapore Health Services Group. This database contains basic demographics of all patients and all mandatory registered visits and admission to our hospitals. Data extracted included age, sex, ethnicity, admission date, discharge date, admission discipline, admission ward class, intensive care unit (ICU) admission, date of emergency department (ED) visits, discharge destination, length of stay (LOS), in-hospital death, and disease codes under ICD-9-AM (International Statistical Classification of Diseases and Related Health Problems, 9th Revision, Australian Modification). We calculated the Charlson Comorbidity Index (CCI) using Deyo’s adaptation [11].

Age, ethnicity, sex and socioeconomic status are risk factors that are known to affect readmissions rate [12,13]. In Singapore, admission ward classes are categorized according to different levels of government subsidies. Ward classes A, B1, B2 and C received a government subsidy of 0%, 20%, 65% and 80%, respectively [14]. In general, patients in the lower socioeconomic groups are more likely to be admitted to beds in wards with a higher level of government subsidy. Therefore, we used admission ward class as a surrogate to measure the socioeconomic status of the patients.

We then determined LACE scores as described by the original authors [7] for all index discharges. The patient cohort was then divided into two groups based on a LACE index cutoff of 10. LACE index values could be calculated as this was a retrospective study, and LACE variables could only be ascertained after patient discharge.

### Study outcomes

The primary outcome of interest was the rate of first unplanned hospital readmission to SGH within 30 days after index discharge. Unplanned readmissions were defined as the first admission to hospital via the ED after the index hospitalization. If a patient visited the ED and was subsequently admitted within 24 hours, this was classified as an unplanned readmission. In our healthcare model, visits to the ED are almost always a result of acute, unanticipated medical events. Planned admissions were defined as any admissions to the hospital without an immediate prior visit to the ED. These were likely to be admissions where arrangements had been made for patients in advance for elective procedures. These planned admissions were usually arranged in the outpatient clinics.

Secondary outcomes were the first unplanned hospital readmission to our hospital within 90 days after index discharge, and the first ED visit to our hospital within 30 days after index discharge (regardless of subsequent hospitalization).

### Data analysis

The primary variable was the LACE index classified as high (≥ 10) or low (< 10). There were no missing data. Other variables analyzed and shown in Table 1 included age group (< 65 or ≥ 65 years), sex, ethnicity, admission ward class, year of discharge, ICU admission, CCI, number of ED visits, and LOS for index hospitalization.

**Table 1:** The demographic, clinical characteristics of hospitalized patients with different risks of readmission

	**LACE < 10, n=107495**	**LACE ≥ 10, n=20055**	**p**^**†**^	
Age, mean year (SD)	52.82 (17.64)	66.46 (14.68)	<0.001	<0.001
Age group, %				
<65 years	72.30	42.08	<0.001	<0.001
≥65 years	27.70	57.92		
Male sex, %	52.52	48.30	<0.001	<0.001
Ethnicity, %				
Chinese	71.20	73.93	<0.001	Reference
Malay	11.61	13.06		0.001
Indian	10.01	9.09		<0.001
Others	7.18	3.91		<0.001
Admission ward class, %				
A	12.49	6.13	<0.001	Reference
B	62.49	56.81		<0.001
C	25.02	37.05		<0.001
Year of discharge, %				
2006	28.00	35.63	<0.001	Reference
2007	22.02	22.26		<0.001
2008	17.40	15.93		<0.001
2009	16.54	13.46		<0.001
2010	16.14	12.73		<0.001
ICU admission, %	0.57	2.35	<0.001	<0.001
Charlson comorbidity index (SD)	0.35 (0.62)	1.64 (0.90)	<0.001	<0.001
No of ED visits in 6 months before the index hospitalization^*^	1.10 (1.10, 1.10)	1.20 (1.20, 1.21)	<0.001	<0.001
Length of stay for index hospitalisation, day^*^	1.91 (1.90, 1.92)	6.21(6.14, 6.29)	<0.001	<0.001

SD, standard deviation. ED, Emergency Department. ^*^Geometric mean (95% confidence interval).

^†^p value was calculated using Chi-Square test except p value for age was calculated using two sample t-test and length of stay using Mann–Whitney U test.

Categorical variables were reported as percentages, and continuous variables as mean and standard deviation with the exception of the number of ED visits in 6 months and LOS where geometric mean and 95% CI were used because of the skewed distribution. Comparison of categorical variables between high and low LACE index groups was performed using the Chi-square test. The number of ED visits in 6 months and LOS were compared using the Mann–Whitney *U* test. Logistic regression was used to assess the association of 30-day or 90-day unplanned readmissions and 30-day ED visits with the LACE index after adjusting for age group, sex, ethnicity, year of discharge, ICU admission and admission ward class. We did not adjust for CCI and number of ED visits to avoid collinearity, as these variables were used to compute the LACE index.

The Hosmer-Lemeshow Chi-square test was used to assess goodness-of-fit of the model. Area under the receiver operating characteristics curve (C-statistic) was used to assess the discriminatory power of the model. All tests were two-sided, with P-values < 0.05 considered statistically significant. Data analysis was performed using STATA Version 10.0 (StataCorp, College Station, TX, USA).

The protocol was approved by the Ethics Committee of SGH. Informed consent was not obtained from the patients as this was exempted by the Ethics Committee.

## Results

Overall, 127 550 patients were eligible for analysis after excluding 5 311 patients. Table 1 shows that patients in the two groups stratified according to LACE index differed significantly with respect to all tested demographic and clinical variables. A total of 20 055 (16%) patients had a LACE index ≥ 10. Patients in this group were older, and a higher proportion were from admission ward class C, had an ICU stay during their index admission, and had high CCI. They also had significantly longer LOS in hospital. These patients also had a higher risk of 30-day unplanned readmission after the index discharge (unadjusted odds ratio [OR]: 4.37; 95% CI: 4.18–4.57) (Table 2). Using the logistic regression model, their risk remained significant after adjusting for age, sex, ethnicity, year of discharge, ICU admission, and admission ward class (OR: 4.88; 95% CI: 4.57–5.22). The goodness-of-fit of the regression model used for 30-day unplanned readmission was assessed with the Hosmer-Lemeshow test (χ^2^ = 13.1, df = 8, P = 0.107). The C-statistic was 0.70, with P < 0.001.

**Table 2:** The rates of unplanned readmissions and 30-days Emergency visits after index discharge the 2 groups of patients

	**LACE < 10, n=107495**	**LACE ≥10, n=20055**	**Unadjusted OR (95% CI)**	**Adjusted OR ( 95% CI)**
30-days unplanned readmission, %	4.87	18.43*	4.37 (4.18-4.57)*	4.88 (4.57-5.22)*
90-days unplanned readmission, %	7.91	29.22*	4.81 (4.61-5.00)*	5.40 (5.11-5.72)*
30-days Emergency visit, %	11.64	27.42*	2.87 (2.77-2.98)*	2.97 (2.81-3.13)*

P value for unadjusted OR was calculated using Chi-Square test; P value for adjusted OR was calculated using Logistic regression with adjustment for age group, gender, ethnicity, year of discharge, ICU admission and admission class. *p<0.001.

Hosmer and Lemeshow test for 30-day all-cause unscheduled readmission, x^2^ = 12.7, df = 8, P = 0.122. C- statistic 0.70; P < 0.001.

The adjusted OR for all the covariates included in the final multivariate logistic regression model for 30-day unplanned readmission after index discharge is shown in Table 3. All variables remained significant in the final model except for ICU admission. However, we did not exclude this variable as we felt that this was a clinically significant variable likely to affect readmission rate.

**Table 3:** Multivariate regression analysis assessing associations of factors with 30-days unplanned readmission after index discharge, n=127550

	**OR**	**95% CI**		**p-value**
**Age group, years**				
<65	Reference			
>= 65	1.73	1.63	1.83	<0.001
**Female sex**	0.85	0.81	0.89	<0.001
**Ethnicity**				
Chinese	Reference			
Malay	0.92	0.86	0.99	0.018
India	1.0	0.93	1.08	0.965
Others	0.77	0.69	0.87	<0.001
**Year of discharge**				
2006	Reference			
2007	0.81	0.76	0.86	<0.001
2008	0.75	0.70	0.80	<0.001
2009	0.74	0.69	0.80	<0.001
2010	0.79	0.74	0.84	<0.001
**ICU admission**	1.14	0.94	1.39	0.176
**Admission class**				
A	Reference			
B	1.83	1.66	2.01	<0.001
C	1.99	1.80	2.20	<0.001
**LACE Group**	4.88	4.56	5.21	<0.001

Logistic regression was used for analysis. OR, odds ratio; CI, confidence interval. Hosmer and Lemeshow test for 30-days unplanned readmission, x^2^ = 12.7, df = 8, P = 0.122. C-statistic 0.70; P < 0.001.

The risk appeared similar at 90 days after index discharge. The unadjusted OR for 90-day unplanned readmission after index discharge was 4.81 (95% CI: 4.61–5.00). The OR with the same adjustment as for 30-day unplanned readmission after index discharge was 5.40 (95% CI: 5.11–5.72). This group of patients was also more likely to visit the ED (unadjusted OR: 2.87; 95% CI: 2.77–2.98; adjusted OR: 2.97; 95% CI: 2.81–3.13).

## Discussion

Our study showed that the LACE index had the ability to discriminate between patients with low and high rates of unplanned readmissions to our hospital. Though the index was developed in Canada, it may be potentially useful for identifying patients likely to have high rates of readmission in a different healthcare setting such as in Singapore. Our first analysis (Table 1) showed that the baseline characteristics of the two groups of patients were very different. We observed that patients with LACE index ≥10 were older and were more likely to have comorbidities. These patients also tended to be from lower socioeconomic classes as they were more likely to be admitted to a class B or C ward. These findings are consistent with our current understanding of the burden and complexity of chronic diseases in the elderly. Such patients are more likely to have limited resources and poor social support, resulting in more complex comorbidities and increased LOS. Providing better social services for this group of patients may be an effective way to reduce unplanned readmissions to hospitals.

We were, however, surprised to find that the risk of patients with LACE index ≥10 for readmission 30 days after the index discharge was five times greater than that of patients with LACE index < 10, even after adjustment. This was higher than was observed in the Canadian study (relative risk: 2.08; 95% CI: 1.95–2.21) [9]. Our 30-day ED visit was also significantly higher compared with the Canadian study (relative risk: 1.27; 95% CI: 1.19–1.35). These differences may reflect the level of primary care and social support that is available to such patients in the two health care systems.

There were several limitations in our study. First, our database did not allow us to account for out-of-hospital deaths. Although the LACE index was originally developed to predict the composite outcome of readmissions and deaths, we were unable to use out-of-hospital deaths and unplanned readmissions as a composite outcome. We tried to address this limitation by excluding patients with in-hospital deaths. Therefore, this study does not allow us to make any conclusion about the performance of LACE in predicting death in our patients.

Second, this was a single center study. We cannot exclude the possibility of patients being readmitted or visiting the ED of other hospitals in Singapore. However, we believe these numbers may be small. Singapore is a small city state and our hospital is centrally located and readily accessible from all parts of the country. Our hospital is a national referral center with a comprehensive range of specialist care services and is more likely to receive patients from other hospitals in Singapore.

Third, in practice, the LACE index cannot be calculated before a patient is discharged. This may limit its practical application. However, this limitation may be overcome by developing a systematic process to calculate the LACE index immediately after discharge for each patient.

Fourth, in the outcome assessment of unplanned readmissions, though most unplanned admissions were through the ED, there may be a small number of unplanned admissions made through outpatient clinics that were not captured.

Finally, as this study did not include surgical patients, the findings of the study cannot be generalized to surgical patients.

## Conclusion

Limitations notwithstanding, we believe that our study has demonstrated the potential application of the LACE index to help identify patients at risk of unplanned readmission in hospitals in Asian countries such as Singapore. Using a LACE cutoff of 10 allowed us to identify a group of patients in our hospital who had a five-fold risk of readmission. This would help us to further examine the reasons for readmission in this group of patients.

Further studies may improve the effectiveness of the LACE index in different healthcare systems. This will involve identifying other factors that may be unique to different countries. Future tools combining the LACE index with additional clinician, system and patient variables may improve its utility.

## Competing interests

The authors declare that they have no competing interests.

## Authors’ contributions

All authors were involved in the conception and design of the study, interpretation of the data, the drafting of the manuscript and the revision of the manuscript. All authors approved the final version submitted for publication. Dr. YY was responsible for the acquisition and the analysis of the data. He also had full access to all data in the study and takes responsibility for the integrity of the data and the accuracy of the data analysis.

## Pre-publication history

The pre-publication history for this paper can be accessed here:

http://www.biomedcentral.com/1472-6963/13/366/prepub
